# Hospital Support for Siblings of Children With Illness in Japan

**DOI:** 10.3389/fped.2022.927084

**Published:** 2022-05-30

**Authors:** Kazuteru Niinomi, Hajime Mihira, Ryota Ochiai, Akiko Misawa, Kuniyoshi Takigawa, Nagako Kashiki, Naoko Kakee, Hidemi Takata, Yasushi Ishida, Takashi Higaki

**Affiliations:** ^1^Department of Integrated Health Sciences, Graduate School of Medicine, Nagoya University, Nagoya, Japan; ^2^Education and Research Center of Legal Medicine, Chiba University, Chiba, Japan; ^3^Department of Nursing, Graduate School of Medicine, Yokohama City University, Yokohama, Japan; ^4^Yamashiro Minami Health Care Center of Kyoto Prefecture, Kizugawa, Japan; ^5^Department of Education, Faculty of Human Development and Education, Kyoto Women's University, Kyoto, Japan; ^6^Graduate School of Education, Ehime University, Matsuyama, Japan; ^7^Division of Bioethics, National Center for Child Health and Development, Tokyo, Japan; ^8^Department of Regional Pediatrics and Perinatology, Graduate School of Medicine, Ehime University, Toon, Japan; ^9^Pediatric Medical Center, Ehime Prefectural Central Hospital, Matsuyama, Japan

**Keywords:** children, chronic illness, hospital support, nationwide survey, siblings, children with illness, Japan

## Abstract

Recent years have seen increased attention to the needs and support of siblings of children with chronic illness, and reports of intervention studies on siblings are gradually increasing worldwide. In Japan, the basic policy approved by the Cabinet in 2021 of The Basic Law for Child and Maternal Health and Development stipulates promoting support for the siblings of children with chronic illness, medical care, and disabilities. Simultaneously, practical reports are emerging. However, reports on the actual state of sibling support at medical institutions in Japan are limited. This study aimed to describe the actual state of support for siblings of children with illness in Japanese medical institutions using a cross-sectional design. Responses were obtained from 207 of 484 registered training facilities for Board-Certified Pediatricians of the Japan Pediatric Society through anonymous questionnaires investigating the actual state of siblings' support. Descriptive statistics were calculated, and the state of siblings' support was described. Fifty-two participants (25.1%) answered that the entire ward, including two outpatient departments, provided siblings' support, while 37 (17.9%) answered some staff made an effort, and 117 (56.5%) did not. Support mentioned included conversing with siblings, actively speaking to siblings, calling siblings' names, and counseling care through the parents. Of the 45 cases (21.7%) where siblings were invited to events and gatherings, 10 (22.2%) were siblings-centered events. Some cases involved collaboration with local sibling support groups such as non-profit organizations. This study clarified the actual state of siblings' support, and further expansion of this support is required.

## Introduction

Recent years have seen increased attention to the needs and support of siblings of children with chronic illness. Since the concerns and attention of parents and other surroundings are primarily directed to children with chronic illness, their siblings are neglected, threatening their self-esteem (1, 2). Siblings also feel guilt about the capacity to lead healthy and enjoyable lives and the perceived pressure to achieve perfection, not be bothered, and suppress their feelings not to burden parents and other caregivers (3–5).

While long-term survival of children with chronic illness has become possible with developing medical care and attention given to health care transition (6), their siblings also face the risk of psychosocial and emotional difficulties, leading to developmental difficulties and concerns beyond the typical adolescence and young adulthood (7). It is crucial to support them in promoting self-reliance from childhood, vital for personality growth, to prevent or reduce these concerns throughout their lives.

Studies report that health-related quality of life (HRQoL) scores of siblings of children with chronic illness were higher than those of children with chronic illness (8). In contrast, HRQoL among siblings of children with chronic illness were reported to be significantly lower than that of healthy children, including mental health aspects, psychosocial aspects, peers, and financial resource dimensions (9, 10), and they are at increased risk of lower self-esteem (2). These concerns arise from family relationships and social interactions such as friends and school. For instance, siblings are at risk of poor academic performance and peer-related challenges at school (11, 12). Further, the family primarily focuses on the ill child; hence, the siblings cannot rely on their family for support and additionally take on household chores responsibilities. Moreover, because of being worried about their ill siblings, they prefer visiting the hospital over being in school and face a lack of concentration in classes.

Siblings need attention and acknowledgment from loved ones and others, instrumental support, and support available specifically for them (13). To meet these needs, appropriate methodologies, evaluation, and psychological wellbeing interventions for siblings of children and young adults with a chronic illness using group study design with developed and reported effects are gradually increasing worldwide (14). Reports of these interventions, including Sibshops and Sibling Coping Together Group, include community-based and hospital-based organizations (3, 15). However, the grasp of the actual situation of sibling support practiced in daily medical care in hospital settings is limited. Furthermore, the actual situation of siblings' support in daily medical care through collaboration with the resources taken by community-based local support groups and hospitals is little known (16).

In hospitals, some children spend a crucial period in treatment to gain physical recovery, while others are eligible for pediatric palliative care that begins at diagnosis (17, 18). While the patient-and family-centered care philosophy is essential for pediatric palliative care (19, 20), the actual care does not include siblings, which is concerning (21).

Healthcare professionals belonging to medical institutions are aware of the need to support siblings; more integration with community services is needed due to the absence of siblings from hospital settings (22). In Japan, the basic policy approved by the Cabinet in 2021 of The Basic Law for Child and Maternal Health and Development stipulates promoting support for the siblings of children with chronic illness, medical care, and disabilities. Furthermore, the Ministry of Health, Labor, and Welfare indicates the need to expand support for siblings, such as implementing self-reliance support projects for specific pediatric chronic illnesses. Moreover, they must cooperate with non-profit organizations (NPOs) and volunteer groups with comprehensive community care. However, actual support for siblings and collaboration with community-based care in medical institutions are little known.

This study aimed to clarify the actual state of support for siblings of children with illness in Japanese medical institutions.

## Materials and Methods

### Design

A cross-sectional study with an anonymous self-administered questionnaire survey was used.

### Participants

Participants were 207 of the 484 registered training facilities for Board-Certified Pediatricians of the Japan Pediatric Society (as of May 13, 2019), whose cooperation was obtained (recovery rate 42.8%).

### Procedure

Based on a list we obtained with the approval of the Japan Pediatric Society, a survey request was mailed to the nursing department directors of 484 registered training facilities for Board-Certified Pediatricians of the Japan Pediatric Society (as of May 13, 2019). The directors were asked to select one chief nurse and hand out the request form and questionnaire. If there were multiple departments engaged in pediatric care, such as the pediatric ward, pediatric outpatient department, and Neonatal Intensive Care Unit (NICU), we requested them to select one participant from one department functioning more or interested in siblings' support. Questionnaires were answered anonymously and retrieved from the participants by mail using a return envelope. The survey period was July–September 2019.

### Questionnaire

Regarding demographics, the survey included details on the type of medical institution, medical service area, number of beds, and departments to which the respondents belonged. For siblings' support, the survey included details on the typically implemented support, whether there are sibling-centered events and content, temporary safekeeping for siblings, method of information-sharing between the staff about the siblings, and implementation of bereavement care. The system for siblings' visitation in the ward and the barriers encountered in the implementation of siblings' support was also included in the survey prepared by the authors for study purposes. All items' implementation status was investigated close-ended by examining whether or not each item was implemented. Besides, free-form answer items were provided in each section, and more specific support content was requested in open-ended questions (Supplementary Material S1).

### Analysis

Statistical analysis was conducted using IBM SPSS Statistics 21 (IBM Corp., Armonk, NY, USA). Of the 484 facilities contacted, 207 responded and returned the questionnaires and were included in the analysis. Efforts were made to understand the status quo by calculating the descriptive statistics of each question.

### Ethical Considerations

This study was conducted with the approval of the Clinical Research and Ethics Review Committee of Ehime University Hospital (No. 1905010).

## Results

### Demographics

The most common medical institution form was “general hospital” (190; 91.8%), followed by “Children's hospital” (10; 4.8%). More than half of medical service areas were “tertiary” (115; 55.6%), followed by “Secondary” (88; 42.5%). Others included an allergy specialty hospital, a rehabilitation hospital, and a treatment and education institution (Table 1).

**Table 1:** Demographics.

**Item**	***n* (%)**
**Form of medical institution**
General hospital	190 (91.8)
Children's hospital	10 (4.8)
Others	7 (3.4)
**Medical service area**
Secondary	88 (42.5)
Tertiary	115 (55.6)
No answer	4 (1.9)
**Department**
Pediatric ward (pediatric internal medicine and pediatric surgery)	118 (57.0)
Pediatric internal medicine ward	25 (12.1)
Pediatric surgery ward	2 (1.0)
Mixed ward for children and adults	48 (23.2)
NICU^+^	9 (4.3)
Outpatient	2 (1.0)
No answer	3 (1.4)

*N = 207*.

+ *Neonatal Intensive Care Unit*.

### Actual Siblings' Support

Regarding actual siblings' support, more than half of the participants subjectively answered, “no efforts are being made on siblings support from staff in the ward (outpatient)” (117; 56.6%), about a quarter answered, “All staff in the entire ward (outpatient) makes an effort” (52; 25.1%), and remaining answered, “some staff in the ward (outpatient) makes an effort” (37; 17.9%) (Table 2).

**Table 2:** Siblings support efforts in the ward.

**Item**	***n* (%)**
The entire ward (outpatient) makes an effort	52 (25.1)
Some staff make an effort	37 (17.9)
No efforts are being made	117 (56.5)
No answer	1 (0.5)

*N = 207 (Including 2 outpatient facilities)*.

Regarding regular direct siblings support provided (multiple answers), 97 (46.9%) answered “conversing with siblings (Medical staff converse with the siblings),” followed by “actively speaking to siblings (Conveying something from medical staff to siblings)” (87; 42%) and “calling siblings by their names” (66; 31.9%) (Table 3). Compared to these three items, the implementation rate of time-consuming interventions such as “playing with siblings” (40; 19.3%) and “explaining the admitted child's medical condition” (29; 14.0%) were low. Many facilities (117) answered “no efforts are being made”; in contrast, 33 (28.2%) answered “conversing with siblings”, 21 (17.9%) answered “actively speaking to siblings”, and 13 (11.1%) answered “calling siblings by their names”.

**Table 3:** Details of siblings' support.

**Item**	***n* (%)**
**Usual direct siblings support (multiple answers)**	
Conversing with siblings	97 (46.9)
Actively speaking to siblings	87 (42.0)
Call siblings by their names	66 (31.9)
Playing with siblings	40 (19.3)
Explaining the admitted child's medical condition	29 (14.0)
Giving medals and autograph cards at discharge	15 (7.2)
Reading and introducing books and picture books concerning the sibling's condition	12 (5.8)
Giving orientation about life when hospitalized	6 (2.9)
Exchanging a diary daily with siblings	2 (1.0)
Others	16 (7.7)
**Explanation of the medical and living conditions of**	
**the child admitted to the hospital**	
All families are asked if they intend to explain to the siblings the situation; the explanation is given based on their decision	8 (3.9)
Explanation given at the family's request	50 (24.2)
Has a track record of providing an explanation in the past	17 (8.2)
No explanation provided	107 (51.7)
No answer	25 (12.2)
**Details of support through parents (multiple answers)**	
Intentionally bringing up siblings in the conversation	124 (59.9)
Providing support when the parent explains the medical condition and hospital stay of the ill child to the siblings	71 (34.4)
Reading and introducing books and picture books concerning the siblings	15 (7.2)
Introducing NPO^+^ activities for siblings' support	7 (3.4)
Others	12 (5.8)
**Events and gatherings to which siblings are invited**	
Yes	45 (21.7)
Siblings-centered events	10 (22.2)
Events centered on pediatric patients	34 (75.6)
No answer	1 (2.2)
No	153 (73.9)
No answer	9 (4.3)
**System for the temporary safekeeping of siblings**	
Yes	20 (9.7)
There are rooms or spaces managed for them in each ward or outpatient department	6 (50)
In one location in the entire medical institution; there is a room or space set aside for siblings	8 (20)
There are no rooms or spaces set aside for siblings	0 (0)
Others	6 (15)
No	184 (88.9)
No answer	3 (1.4)
**Rooms (family room) where the family, including the siblings and**	
**inpatient, can gather together (excluding two outpatient facilities)**	
Yes	27 (13.1)
No	176 (85.9)
No answer	2 (1.0)
**Long-term stay facilities or similar arrangements that the families**,	
**including the siblings, can use**	
There is no facility that can be introduced	143 (69.0)
The facility is inside or in an adjacent location to the medical institution	36 (17.4)
Though not adjacent, there is a facility introduced on a daily basis	16 (6.3)
Others	6 (4.3)
No answer	4 (1.9)

*N = 207*.

+ *Non-profit organizations*.

The highest implementation support was through parents (multiple answers). Furthermore, the highest among all the questions was “intentionally bring up siblings in the conversation” (124; 59.9%). Conversely, implementation of “introducing non-profit organization activities for sibling support” (7; 3.4%) was low (Table 3).

Of the 45 facilities (21.7%) implementing events and gatherings to which siblings are invited, the forms of implementation were “events centered on pediatric patients” for 34 facilities (75.6%) and “events centered on their siblings” for 10 facilities (22.2%) (Table 3). Of these 10 facilities, no events were held for more than 15 years, and the maximum was “since over 10 years ago” for two facilities (20%); the starting period for most of them was “since over 1 year to at most 5 years” for five facilities (50%). Additionally, event frequency ranged from “six times a year” for one facility to “once a year” for one facility (10%).

There were 20 (9.7%) who answered “Yes” for siblings' temporary safekeeping. Of these 20, six (15%) responded “Others”, unspecific locations such as staff or nurse stations, lounges, and conference rooms were used. Additionally, for the frequency of opening the rooms for siblings' safekeeping, ten (50%) answered “every day”, while three (15%) answered “not regularly opened”; for a staff member being present continuously, six (30%) answered “Yes”. Furthermore, for efforts made regarding rooms and space (multiple answers), 13 (65%) answered “playing with cards and toys”, 11 (55%) answered “conversing with the siblings”, and 5 (25%) answered “working on the sibling's homework together”.

### Bereavement Support for Siblings

Regarding bereavement support for the siblings, while the response “support efforts are made when death approaches” was the most frequent at 56 (27.1%), 23 (11.11%) answered, “pediatric palliative care, including siblings, is implemented from the time of diagnosis”, and 15 (7.2%) answered, “there are support efforts post-bereavement” (Table 4). The result from the answers obtained in the free-form description by the respondents of their specific efforts was, “becoming involved with siblings from the time of the pediatric patient's admission makes it easier to provide support even when the patient's condition deteriorates and accept consultation from the parents”, suggesting the necessity of interacting with siblings form an early stage. Besides, some facilities also cooperated with local support groups: “we sometimes collaborate with the local community,” and “we provide information to the non-profit organization and have them participate in expansion conferences”. Further, among the free responses regarding post-bereavement, one responded that “we interact with the school,” and another, “we are currently planning a grief workshop with siblings”.

**Table 4:** Bereavement support for siblings (multiple answers).

**Item**	***n* (%)**
Pediatric palliative care, including siblings is implemented from the time of diagnosis	23 (11.1)
Support efforts are made when death approaches	56 (27.1)
There are support efforts for siblings' post-bereavement	15 (7.2)

*N = 207*.

### Efforts Made to Share Siblings-Related Matters Among the Staff

Regarding efforts made to share siblings-related matters among the staff, 80 (11.1%) responded “Yes” (Table 5). Of these 80 responses (multiple answers), the majority at 59 (73.8%) answered they “share the status of siblings formally, such as in conferences”.

**Table 5:** Efforts made to share sibling-related matters among the staff.

**Item**	***n* (%)**
Yes, there are instances (multiple answers, *n* = 80)	80 (38.6)
The status of siblings is shared formally, such as in conferences	59 (73.8)
The status of siblings is described and shared in the medical record	41 (51.3)
Staff talk regularly about the siblings' status on a daily basis	34 (42.5)
Others	0 (0)
No	115 (55.6)
No answer	12 (5.8)

*N = 207*.

### Visitation Between Inpatients and Siblings in the Ward

In results obtained from the responses of 205 facilities, excluding two outpatient facilities, regarding visitations between siblings and inpatients, the majority stated that “visitation has restrictions” (195; 94.6%) (Table 6). The details about the restrictions of these 195 facilities concerning age, health condition, and siblings' visitation time are as follows. For siblings' age, most visitations in the ward were limited to “high school and above” with 70 (35%), while 37 (19%) had “no age restrictions”. For restrictions on visitation depending on the siblings' health condition, 28 facilities (15.3%) “confirm the sibling's vaccination status with the Maternal and Child Health Handbook”, while most were “self-declaration of the siblings or siblings' parents” (147; 80.3%). For the 164 (84.1%) who responded “Yes” to visitation restrictions based on time, most answered that visits were possible between ~13:00 and 20:00 (UTC+9).

**Table 6:** Visitation between inpatients and siblings in the ward.

**Item**	***n* (%)**
**Visitation has restrictions** **Visiting restrictions based on the age of siblings (*****n*** **=** **195)**	195 (94.6)
High school and above allowed	70 (35.9)
Junior high school and above allowed	40 (20.5)
Elementary school and above allowed	13 (6.7)
Two years old and above allowed	1 (0.5)
Others	26 (13.3)
No age restrictions for siblings	37 (19.0)
No answer	8 (4.1)
**Visiting restrictions based on sibling's health condition (*****n*** **=** **195)**	
Yes	183 (93.8)
**Method to confirm health condition (multiple answers**, ***n*** **=** **183)**	
Confirming vaccination status with the Maternal and Child Health Handbook	28 (15.3)
Confirming the infection trend at the nursery school or school	79 (43.2)
Confirming the infection trend in the area where the sibling lives	13 (7.1)
Self-declaration by the sibling or parents regarding health condition and signs of infection on that day	147 (80.3)
The medical professionals confirm the health condition and signs of infection on that day	68 (37.2)
Others	16 (8.7)
No	8 (4.1)
No answer	4 (2.1)
**Visiting restrictions based on time (*****n*** **=** **195)**	
Yes	164 (84.1)
Visitation is allowed 24 h a day	16 (8.2)
No answer	15 (7.7)
**Visitation without restrictions is allowed for siblings of the patient**	9 (4.9)
**No answer**	1 (0.5)

*N = 205 (excluding two outpatient facilities)*.

### Barriers to Implementing Siblings Support

Regarding barriers to implementing siblings support (multiple answers), 136 (65.7%) responded with “Human resources” and “time” 102 (49.3%), while those who responded with “others” 63 (30.4%) specified the following barriers: risk of infection and lack of space and staff awareness (Table 7).

**Table 7:** Barriers to implementing siblings support (multiple answers).

**Item**	***n* (%)**
Time	102 (49.3)
Human resources	136 (65.7)
Other barriers	63 (30.4)
Cannot sense any barriers	17 (8.2)

*N = 207*.

## Discussion

Since this survey results were obtained from over 40% of the hospitals registered as training facilities for Board-Certified Pediatricians of the Pediatric Society, it may approximately reflect the actual situation of siblings support at facilities representing pediatric practice in Japan. Wiener et al. (23) reported that pediatric psycho-oncology professionals from 63 countries responded that 25% provide individual psychosocial sessions with siblings and 6% provide sibling group sessions. Since this study is not limited to pediatric oncology, it is impossible to make a direct comparison; however, it is expected that the actual situation related to siblings in Japan is not high.

The survey results indicate that even facilities that responded with no efforts toward siblings' support perform actions such as conversing with siblings based on the specific details they provided. Many siblings valued personal contact with physicians and nurses at the hospital and mainly described hospital staff as friendly, helpful, and willing to inform them about the illness (24). Moreover, in Japan, adolescent siblings of children with chronic illnesses answered, “I was happy that medical staff remembered my name” and “The hospital staff spoke to me in the corridor, then we enjoyed playing with cards or some…it was a fun time” (25). Therefore, individual awareness from medical staff such as these actions leads to support and helps establish good relationships with siblings.

Siblings have a strong desire to know and understand their ill sibling's condition. Moreover, information helps siblings' engagement with family and familiarity with hospital environments (26). Moreover, interaction with medical care, system, and staff influence siblings (27). In hospital settings, siblings of admitted children are specifically at risk of unhealthy psychosocial conventions, such as internalizing distress (27). Further, siblings attempt to maintain their usual self, an essential element of children's optimal growth and development, while controlling for internalizing and externalizing complexities (5). However, siblings are largely absent from family-centered care and pediatric healthcare settings (21). Moreover, siblings are often overlooked by clinical staff due to a lack of apparent features in hospital settings (22). To grasp admitted children's siblings' condition and promote appropriate support, healthcare professionals must communicate and build good collaborative relationships with their parents and other caregivers (5). It is challenging for medical professionals to build good relationships with parents for siblings' support. Moreover, pediatric hospitalization causes psychological distress to their siblings and parents (28).

Some siblings ask for information and believe it should be provided continuously by the healthcare providers (29). Nevertheless, the result indicated that most facilities did not explain the medical and living conditions of the admitted child directly to their siblings. In contrast, information is often directly provided to siblings by their parents (26). Additionally, more than half of the participants in this study answered that they intentionally bring up siblings in conversation with their parents to provide indirect siblings support. Meanwhile, intervention programs for siblings are increasing (30), involving parents for support, and parent-child communication intervention studies are gradually increasing (31, 32). Moreover, siblings desire honest and open family communication (13). Hence, highly accurate programs are required to meet the needs of siblings and their parents that can easily be used by medical professionals at hospitals.

Community resource availability is essential for providing psychosocial support for siblings (33). The study results indicated that few facilities introduce parents to NPO activities for siblings' support. Moreover, some facilities reported collaborating with the local community in the bereavement care process. This collaboration may help with continued support to reach siblings even after the ill child is discharged from the hospital. Integrating hospital-based care with community services may better facilitate the engagement and support of siblings (22). Further, the collaborative approach between hospital-and community-based support may provide psychosocial support to siblings of youth with chronic illnesses (16). In Japan, within the caregiver support project, a self-reliance support project for children with specific pediatric chronic illnesses whose implementing bodies include prefectures, the necessity of siblings' support is firmly stated as safekeeping support for siblings. One recommended project endeavor is to collaborate with specified NPOs and volunteer groups. From this, establishing a collaboration system among prefectures, support groups, and medical institutions may enhance siblings' support circumstances.

School-based social support is valued and related to siblings' emotional, behavioral, and academic adjustments (34). Moreover, Gerhardt et al. (33) suggested partnering with other professionals (such as teachers and community-based providers) to anticipate and address siblings' psychosocial needs. Flexibility in location and care modality is often necessary as contact with siblings may be restricted due to hospital policy or for practical reasons. This is especially true after a child's death. In this study, one responded that they interact with the school about siblings in the post-bereavement process. However, few studies have examined the effect of teachers or peers in providing social support for siblings (11). Siblings give importance to the relationship with teachers in school and their friends. Collaboration between medical professionals such as designated nurses and schools is essential to meet these siblings' needs and implement family-centered care (35, 36). Further collaboration between hospitals and schools must be promoted in the future.

In the United States, to maintain the balance between promoting family-centered care and infection control, the restriction on siblings <12 years old from visiting hospitalized children until the 1980s is now alleviated, and they are encouraged to visit (37). Hence, the benefits of visiting admitted children with chronic illness for their siblings have been reported and well known since (38). In contrast, there are still visitation restrictions for even 13 years above age siblings in Japan to prevent infection. The reasons for these restrictions, other than to prevent infections, are not well known or studied. Despite the benefit and protection of siblings visiting and helping their brother or sister in the hospital (21, 38), the result indicated that almost all facilities have visitation restrictions imposed on siblings' age, health condition, and visitation period. This restriction creates a distance between siblings and medical care; however, the actual situation has not been previously clarified. Moreover, the regulations vary depending on the facility, and no provision as a gold standard. Although there is no clear evidence, many Japanese hospitals have traditionally placed visit restrictions to prevent infectious diseases. A report showed that siblings' visits to the NICU did not increase the viral infection rate (39); therefore, appropriate visitation restrictions must be lifted while investigating verification under various conditions such as pediatric wards. Moreover, these restrictions must be explained, and methods should be considered to better deal with siblings' desire to visit.

Additionally, “staff awareness” was answered in the free-form description; the most rated barrier to implementing sibling support was human resources in this study. Simultaneously, the availability of trained psychosocial staff, staff knowledge concerning siblings, and healthcare providers' access and communication with siblings were barriers to supporting siblings (33). Considering that limited time is subsequent to human resources in the results regarding barriers, sharing siblings matters in daily medical care is required to adequately support staff's learning about siblings issues and practice its implementation.

### Limitations

This survey data was obtained with the consent and free will of each facility. The institutions that do not support siblings may not have been interested in this survey or chose not to disclose their information. Further, if there were multiple departments, we requested selecting one that functions more or is interested in siblings' support. Therefore, the results may have reflected facilities more inclined toward siblings' support. Nevertheless, the results can be used for further developing a siblings' support system.

In this study, only descriptive statistical results were used to represent the situation of siblings' support in the overall medical institutions. The differences in support depending on the attributes must be analyzed and clarified, including between forms of the medical institution, medical service area, or department types, using inferential statistics.

Siblings' developmental stages (such as preschool-age, school-age, and adolescence or young adulthood) are critical for planning and implementing support (40). Siblings are affected by their brother or sister's type of disease and condition, and the hospital-based approach also has a characteristic influence (9). However, this survey did not analyze whether there are differences in the developmental stage of the siblings or the type of illness or condition of their brother or sister.

While there are studies evaluating the effects of educational interventions of a few days or conducting camps for siblings (14), it is not easy to assess the impact of daily involvement as investigated in this study. Additionally, this study is novel since the influence of pediatric medical care involvement on the siblings transitioning from childhood to adolescence and adulthood has not been previously verified. Future studies must evaluate the effect of actual siblings' support using a long-term longitudinal or retrospective study.

## Conclusion

This study clarified the actual state of siblings' support and showed the need for improvements and further expansion of this support in Japan. Some cases involved collaboration with local sibling support groups, such as non-profit organizations and a school.

While it is necessary to raise awareness of medical professionals regarding sibling support, collaboration with hospital-based care, community resources such as local siblings support groups, and school-based social support facilitates engagement and meets siblings' needs. These collaborations may help with continued support to reach siblings even after the ill child is discharged from the hospital, transitioning into adult care, or the contact with medical care becomes low in the future.

## Data Availability Statement

The raw data supporting the conclusions of this article will be made available by the authors, without undue reservation.

## Ethics Statement

This study was conducted with the approval of the Clinical Research and Ethics Review Committee of Ehime University Hospital (No. 1905010). The patients/participants provided their written informed consent to participate in this study.

## Author Contributions

KN, HM, and TH conceived of the present idea. KN, HM, RO, AM, KT, and TH designed this study, performed the survey, and collected and analyzed the data. KN and HM interpreted and analyzed the data. KN drafted the manuscript. HM, RO, KT, NKas, NKak, HT, YI, and TH supervised the entire study process. All authors read and approved the final manuscript.

## Funding

This work was supported by the Ministry of Health, Labor and Welfare Intractable and Rare Disease Program Grant Number JPMH18FC1017.

## Conflict of Interest

The authors declare that the research was conducted in the absence of any commercial or financial relationships that could be construed as a potential conflict of interest.

## Publisher's Note

All claims expressed in this article are solely those of the authors and do not necessarily represent those of their affiliated organizations, or those of the publisher, the editors and the reviewers. Any product that may be evaluated in this article, or claim that may be made by its manufacturer, is not guaranteed or endorsed by the publisher.
