# Supplementary material for: Hospital Support for Siblings of Children With Illness in Japan
Source: Front Pediatr. 2022 May 30;10:927084. doi: 10.3389/fped.2022.927084 (PMC9195589; doi:10.3389/fped.2022.927084)
Supplement: Supplementary file 1 [file Data_Sheet_1.PDF]

Introduction

If you agree to participate in this questionnaire survey, please mark ☑ in the box below.

□ I consent

Section I Answer the following questions on your affiliated medical institution or department.

Please put mark (☑) to indicate your answer to the following questions.
As applicable, please provide appropriate numbers and description (within parentheses).

1 Type of medical institution you are affiliated with (mark ☑ one)

□ General hospital □ Children's hospital □ Others ( )

2 Level of the medical institution you are affiliated with (mark ☑ one)

□ National (including independent administrative agencies) □ Prefectural (including independent administrative agencies)

□ Municipal (including independent administrative agencies) □ Medical institutions (private)

3 Your main medical service area (mark ☑ one)

□ Secondary □ Tertiary

※Secondary: A local core medical institute responsible for the care of critically ill patients requiring inpatient treatment.

※Tertiary: Medical institutions that treat critically ill patients who cannot be treated at a secondary medical institution and provide advanced medical care

4 The main department you are affiliated with (mark ☑ one)

□ Pediatric internal medicine ward □ Pediatric surgery ward

□ Pediatric ward (pediatric internal medicine and pediatric surgery)

□ Mixed ward for children and adults □ NICU □ PICU/HCU □ Outpatient

Please provide more specific information on the ward or outpatient department within the parentheses.

e.g., pediatric internal medicine, hematology, schoolchildren / adolescent surgery and internal medicine mixed wards, discharge support wards, orthopedics, neurosurgery, otolaryngology, etc.

( )

5 Total number of hospital beds (number of hospital beds permitted) in the medical institution you are affiliated with (mark ☑ one)

□ 99 or less □ 100 to 399 beds □ 400 to 999 beds □ 1,000 beds or more

6 Number of beds in the department you are affiliated with

( ) beds

7 Number of nurses in the department you are affiliated with (including assistant nurses)

( ) nurses

Section II These questions are about the "system" used in your ward.

<u>If you work for an outpatient department, please proceed to Section III.</u>

Please put mark (☑) to indicate your answer to the following questions.
As applicable, please provide appropriate numbers and description (within parentheses).

1 Visitation of the inpatient's siblings in the ward (mark ☑ one)

□ Visitation has restrictions **⇒ Proceed to 2**

□ No restrictions **⇒ Proceed to 10**

Question 2 is to be answered by those who marked ☑ "Visitation has restrictions" for Question 1.

2 Visitation restrictions based on the age of siblings (mark ☑ one)

□ Restrictions apply based on the age of the sibling

□ No age restrictions **⇒ Proceed to 4**

3 Age at which siblings can visit (mark ☑ one)

□ High school or above

□ Junior high school or above

□ Elementary school or above

□ Two years old or above

□ Others ()

4 Visitation restrictions based on the sibling's health condition (mark ☑ one)

□ Restrictions apply based on sibling's health condition.

□ No restriction based on sibling's health condition. **⇒ Proceed to 7**

5 Aspects related to health condition to confirm (mark ☑ all that apply)

□ Vaccination status in the Maternal and Child Health Handbook

□ The infection trend at the nursery school or school

□ The infection trend in the area where the sibling lives

□ Self-declaration by the sibling or parents regarding health condition and sign of infection on that day

□ Confirmation of health condition and sign of infection on that day by medical professionals

6 Describe as specifically as possible within [] about the content marked ☑ in 5 above.

[e.g., For outpatients, body temperature is measured by a nurse, and signs of infection are checked using a questionnaire.
Check the vaccination status based on age according to the Maternal and Child Health Handbook, etc.]

7 Visiting restriction based on time (mark ☑ one)

□ Visitation is allowed 24 hours a day

□ Restrictions apply by visiting time (Time at which visiting is allowed:)

8 Restrictions other than age, health condition, and visiting hours (mark ☑ one)

□ Other restrictions and conditions apply

□ No other restrictions or conditions **⇒ Proceed to 10**

9 Describe specifically the restrictions in 8 above.

[e.g., During the terminal phase, up to two family members are allowed to enter the ward in a day.]

10 Rooms (family room) where the family, including the siblings and the inpatient, can gather together

□ Rooms (family rooms) available for the gathering of family members.

□ None **⇒ Proceed to 12**

11 Describe specifically whether restriction/conditions apply for using the family rooms in 10 above.

[For example, "age restriction" and "checking the health status of a sibling" are the same as those that apply for visiting,
and "visit time" is restricted to 9:00-16:00, etc.]

12 Long-term stay facilities or similar arrangement that the families including the sibling can use (mark ☑ one)

□ There is no facility that can be introduced

□ Facility is inside or in an adjacent location to the medical institution

□ Though not adjacent, there is a facility introduced on a daily basis

□ Others (Please describe specifically:)

Section III Please tell us about the actual status of "support for siblings" in your department.

Please mark (☑) in the one applicable for each of the following questions.

As applicable, please provide appropriate numbers and description (within parentheses).

1 How do you engage in "siblings support effort in the ward (outpatient)" in your ward or outpatient department? (mark ☑ one)

□ The entire ward (outpatient) makes an effort

□ Some staff make an effort

□ No efforts are being made

2 What kind of "usual direct sibling support" are you providing? (mark ☑ all that apply)

□ Actively speaking to siblings

□ Calling siblings by their name

□ Conversing with siblings

□ Playing with siblings

□ Giving orientation about life when hospitalized

□ Explaining the admitted child's medical condition

3 System for the temporary safekeeping of siblings (mark ☑ one)

- □ System for the temporary safekeeping of siblings
- □ No system in place for the temporary safekeeping of siblings **⇒ Proceed to 10**

4 Rooms and spaces where the siblings can stay while under custody (mark ☑ one)

- □ There are rooms or spaces managed for them in each ward or outpatient **⇒ Proceed to 5** **⇒ Proceed to 5**
- □ In one location in the entire medical institution; there is a room or space set aside for siblings **⇒ Proceed to 5**
- □ There are no rooms or spaces available for siblings **⇒ Proceed to 10**
- □ Others ()

Questions to be answered for those who have marked ☑ against "There are rooms and spaces managed in each ward or outpatient department" in Question 4

5 What are the types of rooms and spaces where a sibling can stay (please describe specifically)?

e.g., There is space to place a mat in the passage outside the ward entrance.

6 Frequency of opening the rooms and spaces in Question 5 above (mark ☑ one)

- □ Every day
- □ Weekdays only
- □ Holidays only
- □ Open regularly

Describe the specific frequency.

- □ Open irregularly (open criteria)

Describe the specific open criteria.

7 Indicate the time when the room or space is open.

e.g., 9:00 to 16:30

8 Are there any permanent staff stationed in the room or space (mark ☑ one)?

- □ Resident personnel (please indicate the occupation of the staff: ________________________________)
- □ None

9 Initiatives in rooms and spaces for siblings visiting (mark ☑ all that apply)

- □ Playing with toys, cards, etc.
- □ Reading a picture book or listening to its narration
- □ Doing homework together
- □ Engaging in conversation
- □ Other

Please describe specifically

10 Do you have "Events and gatherings to which siblings are invited" (mark ☑ one)?

- □ No
- □ Yes

11 What are the type of events? (mark ☑ one)

- □ Pediatric patients-centered events **⇒ Proceed to 15**
- □ Siblings-centered events

Please describe in details

12 Please specify the details of siblings-centered events.

13 Please indicate the timing and frequency of siblings-centered events.

◆Timing: (   )

◆ Frequency: (   ) times a year

14 When did holding the sibling-centered events start? (mark ☑ one)

□ About one year ago

□ One to five years ago

□ More than ten years ago

□ More than 15 years ago

15 Have the healthcare professionals provided "Explanation of the medical and living conditions of the child admitted to the hospital"? (mark ☑ one)

□ All families are asked if they intend to explain to the siblings about the situation; the explanation is given based on their decision

□ Explanation given at the family's request

□ Availability of a track record of providing explanation in the past

□ No explanation provided   **⇒ Proceed to 17**

□ Others

Please describe specifically

16 Please describe the considerations and contrivances adopted when explanations from healthcare professionals are provided to siblings.

17 Details of support through parents (mark ☑ all that apply)

□ Intentionally bringing up siblings in the conversation

□ Providing support to the siblings when the parent explains the medical condition and hospital stay of the ill child

□ Reading and introducing books and picture books concerning the siblings

□ Introducing non-profit organizations activities for siblings support

□ Others

Please describe specifically.

18 For jobs that are directly related to siblings (mark ☑ all that apply)

□ Doctor □ Nurse □ Nursery teacher □ School teacher

□ Nursing assistant □ Volunteer □ Pharmacist □ Psychologist

□ Social worker □ Occupational therapist □ Physical therapist □Speech therapist

□ Child life specialist (CLS) □ Hospital play specialist (HPS)

□ Child care staff (CCS) □ Radiologist □ Inspection engineer □ Clerk

□ Cleaning staff □ Security guard

□ Siblings support team-like organization

Please describe specifically the constituent types of occupations and roles.

□ Other ( )

19 Efforts made for sharing sibling-related matters among the staff (mark ☑ one)

□ Yes, there are instances

□ Have not been addressed   **⇒ Proceed to 21**

20 How do you share the information? (mark ☑ one)

- □ The status of siblings is shared formally, such as in conferences.
- □ The status of siblings is described and shared in the medical record.
- □ Staff talk regularly about the siblings' status on a daily basis.
- □ Others

(Please describe specifically.)

21 Bereavement support for siblings (mark ☑ all that apply)

- □ Pediatric palliative care including siblings is implemented from the time of diagnosis

(Please describe specifically.)

- □ Support efforts are made when death approaches

(Please describe specifically)

- □ There are support efforts for siblings' post-bereavement

(Please describe specifically)

Section IV Please tell us about your thoughts/perceptions.

Please mark (☑) in the one applicable for each of the following questions.

As applicable, please provide appropriate numbers and description (within parentheses).

1 Thoughts about "barriers to implementing sibling support"

- □ Time
- □ Human resources
- □ Other barriers
- □ Cannot sense any barrier

(Describe specifically the perceived barrier.)

2 Please describe the sibling support-related activities you have devised or valued so far, if any.

( )

You have reached the end of the survey.
Thank you very much for your cooperation despite your busy schedule.
Your cooperation is much appreciated.
